# Avian Tembusu virus infection effectively triggers host innate immune response through MDA5 and TLR3-dependent signaling pathways

**DOI:** 10.1186/s13567-016-0358-5

**Published:** 2016-07-22

**Authors:** Shilong Chen, Guifeng Luo, Zhou Yang, Shuncheng Lin, Shaoying Chen, Song Wang, Mohsan Ullah Goraya, Xiaojuan Chi, Xiancheng Zeng, Ji-Long Chen

**Affiliations:** College of Animal Sciences, Fujian Agriculture and Forestry University, Fuzhou, 350002 China; College of Life Sciences, Fujian Agriculture and Forestry University, Fuzhou, 350002 China; CAS Key Laboratory of Pathogenic Microbiology and Immunology, Institute of Microbiology, Chinese Academy of Sciences (CAS), Beijing, 100101 China; Institute of Animal Husbandry and Veterinary Medicine, Fujian Academy of Agriculture Sciences, Fuzhou, 350002 China

## Abstract

Avian Tembusu virus (ATMUV) is a newly emerged flavivirus that belongs to the Ntaya virus group. ATMUV is a highly pathogenic virus causing significant economic loss to the Chinese poultry industry. However, little is known about the role of host innate immune mechanism in defending against ATMUV infection. In this study, we found that ATMUV infection significantly up-regulated the expression of type I and type III interferons (IFN) and some critical IFN-stimulated genes (ISG) in vivo and in vitro. This innate immune response was induced by genomic RNA of ATMUV. Furthermore, we observed that ATMUV infection triggered IFN response mainly through MDA5 and TLR3-dependent signaling pathways. Strikingly, shRNA-based disruption of IPS-1, IRF3 or IRF7 expression significantly reduced the production of IFN in the 293T cell model. Moreover, NF-κB was shown to be activated in both chicken and human cells during the ATMUV infection. Inhibition of NF-κB signaling also resulted in a clear decrease in expression of IFN. Importantly, experiments revealed that treatment with IFN significantly impaired ATMUV replication in the chicken cell. Consistently, type I IFN also exhibited promising antiviral activity against ATMUV replication in the human cell. Together, these data indicate that ATMUV infection triggers host innate immune response through MDA5 and TLR3-dependent signaling that controls IFN production, and thereby induces an effective antiviral immunity.

**Electronic supplementary material:**

The online version of this article (doi:10.1186/s13567-016-0358-5) contains supplementary material, which is available to authorized users.

## Introduction

Avian Tembusu virus (ATMUV), a newly emerged flavivirus, is the causative agent of acute egg-drop syndrome in domestic poultry of China since 2009 [1–4]. Clinical symptoms of the infected birds are characterized by anorexia, ataxia and abrupt drop in egg production [1–4]. Similar symptoms have been reported in young Pekin ducks from Malaysia, where infected birds showed neurological disorders, including ataxia, lameness and paralysis in year 2012 [5]. To date, ATMUV infection has been confirmed in ducks, chickens and geese, and it causes significant economic losses to the poultry industry in China. In addition, a number of humans have been found to be positive for high levels of serum-neutralizing antibodies against Tembusu virus, suggesting that this virus has zoonotic potential [6]. Moreover, RNA of ATMUV and neutralizing antibodies had also been detected in duck farm workers in Shandong, China [7]. This evidence suggests that ATMUV could be a threat to farm workers. Despite its zoonotic risk, no commercial vaccine or specific therapy has been developed to prevent and control the ATMUV infection. Importantly, pathogenesis of ATMUV is still not fully understood.

The host innate immune system provides the first line of defense against pathogens, which is the more rapid immune response but lacks memory and specificity as compared to adaptive immunity [8, 9]. Host cells recognize the pathogens by sensing the different molecules or structure of the pathogen, which are known as pathogen associated molecular patterns (PAMP) via pattern recognition receptors (PRR). Such receptors include Toll-like receptors (TLR), the RIG-I like receptors (RLR) and NOD like receptors (NLR) [10]. To date, 13 TLR in mammals and 10 TLR in chickens have been identified [11, 12]. RLR comprise three helicases: RIG-I, melanoma differentiation associated protein 5 (MDA5), and laboratory of genetics and physiology 2 (LGP2) [8, 13, 14]. Upon sensing viral infection, particular PRR that contain caspase-recruiting domains (CARD), interacts with interferon-β promoter stimulator-1 (IPS-1, also known as VISA, MAVS or Cardif) through CARD–CARD interaction. This interaction activates members of the IKK protein kinase family [15, 16]. The canonical IKK family members IKKa and IKKb mediate the phosphorylation and degradation of I-κB, an inhibitor of NF-κB, leading to activation of NF-κB. The non-canonical IKK family members TBK1 and IKBKE activate the interferon regulatory factor 3 (IRF3) and IRF7 to form a functional homodimer or heterodimer. Thus, the transcription factors IRF and NF-κB translocate to the nucleus to stimulate expression of interferon (IFN) and pro-inflammatory cytokines [16–18]. IFN induce the downstream synthesis of hundreds of antiviral proteins encoded by IFN-stimulated genes (ISG). Various ISG proteins such as IFIT, IFITM, Mx1 and OASL, play key roles in host immune defense against viral infections [19–21]. Therefore, the IFN-activated signaling pathway is an important component of the innate immune system and has been implicated in clinical antiviral treatment [22].

There are three distinct interferon families that have been identified in both mammalian and avian species: type I IFN, type II IFN and type III IFN [23]. Type I IFN is comprised of IFN-α and IFN-β; while the type II is comprised of IFN-γ only. The recently classified type III IFN is comprised of IFN-λ (lambda) which consists of three members named as IFN-λ1, IFN-λ2 and IFN-λ3 (also called IL-29, IL-28A and IL-28B, respectively) [24, 25]. However, only one IFN-λ gene appears to exist in chickens [23]. Type I and type III IFN are the principal cytokines that mediate early antiviral responses, whereas type II IFN produced by T cells and NK cells is an important regulator of cellular immunity and is a classical regulator of Th1 immunity [26]. It is well known that expression of type I IFN is regulated through two phases during viral infection. At the early phase of viral infection, phosphorylated IRF3 and IRF7 translocate to the nucleus and trigger the expression of small amounts of early IFN-β and IFN-α. In the second phase of infection, robust transcription of IFN genes is induced and newly synthesized IFN bind to the type I IFN receptor (IFNAR) and activate the JAK/STAT pathway, leading to the up-regulation of hundreds of ISG [27–29]. These antiviral components inhibit viral replication and cause apoptosis of infected cells, subsequently resulting in the clearance of the infectious pathogens [30]. However, precise mechanisms underlying interaction between host innate immune system and numerous viruses including some flaviviruses are still not fully understood [31–34].

The flaviviruses express two key PAMP: one is the genomic ssRNA of the virus and the second is dsRNA replication intermediates. It has been previously shown that RLR and TLR3, 7 and 8 are involved in sensing the RNA viruses [10, 13, 35]. Recently, innate immune response to some Flavivirus infections have been studied, such as innate immunity against Dengue virus, Japanese encephalitis virus, and West Nile virus [15–17, 19, 34–37]. However, little information is available on the role of the innate immune system in the control of ATMUV infection. In this study, we investigated the innate immune signaling relevant to the host response against ATMUV infection. We found that ATMUV infection resulted in significant up-regulation of mRNA levels of type I and type III IFN in vivo and in vitro mainly through MDA5 and TLR3 dependent signaling pathways. Disrupting the expression of PRR, IPS-1, IRF3, IRF7 and suppressing NF-κB significantly inhibited the production of IFN-β, IL-28A/B and IL-29 in the host following ATMUV infection. These results reveal that ATMUV infection can activate host innate immune signaling pathways that govern IFN-mediated antiviral immune response.

## Materials and methods

### Ethics statement

The animal protocol used in this study was approved by the Research Ethics Committee of the College of Animal Science, Fujian Agriculture and Forestry University (Permit Number PZCASFAFU2014002). All chicken experimental procedures were performed in accordance with the Regulations of the Administration of Affairs Concerning Experimental Animals approved by the State Council of China.

### Reagents

The antibodies used in this study are described as follows: Mouse Anti-β-actin (ab8226, Abcam, Cambridge, UK), Rabbit anti-IKBα (ZS3710, ZSQB-BIO, Beijing, China), HRP Goat anti-Rabbit IgG antibody (LP1001a, ABGENT, USA) and HRP Goat anti-Mouse IgG antibody (LP1002a, ABGENT). The pharmacological NF-κB inhibitor BAY11-7082 was purchased from Merck (Darmstadt, Germany). Recombinant human IFN-β was purchased from Pepro-Tech (Rocky Hill, NJ, USA). Avian IFN were purchased from Dalian Sanyi Animal Medicine Co. Ltd (Dalian, China). Lipofectamine 2000 was obtained from Invitrogen (Carlsbad, CA, USA).

### Cell lines, birds, virus and infection

Chicken embryo fibroblasts (CEF) were prepared from 11 day-old SPF chicken embryo as previously described [38]. 293T cells were purchased from American Type Culture Collection (Manassas, VA). Both CEF and 293T cells were cultured at 37 °C with 5% CO_2_ in DMEM (Sigma, USA) supplemented with 10% fetal bovine serum (FBS, HyClone, Logan, Utah, USA). ATMUV strain CJD05 used in this study was previously isolated from naturally infected egg-laying fowl in China which shares 98.3–99.3% complete genome homology with waterfowl ATMUV [1]. Cells were infected with CJD05 and incubated for 1 h at 37 °C. Then the cells were washed once with phosphate-buffered saline (PBS) and cultured in DMEM supplemented with 2% FBS at 37 °C with 5% CO_2_ for 3–4 days. Three further passages of ATMUV in 293T and CEF were done using cell suspensions from the previous passage. 293T and CEF cells were infected with the 4^th^ passage virus at the multiplicity of infection (MOI) of 1.0 and harvested at different time points (0–42 h in CEF and 0–48 h in 293T cell) post infection. Five day old specific pathogen-free (SPF) chicks (Harbin Veterinary Research Institute, Chinese Academy of Agricultural Sciences) were challenged with 0.4 mL of CJD05 (the 5^th^ passage allantoic fluid virus, ELD_50_ = 10^−6.0^/mL) per chick by intramuscular injection. Before and post infection, three chicks were sacrificed per day and the spleens were harvested for further examination.

### Viral genomic RNA and viral RNA preparation and their transfection

The ATMUV genomic RNA (VG RNA) was extracted from the purified virus particles using EasyPure Viral RNA Kit (TransGen Biotech, Beijing Co., Ltd) according to the manufacturer’s instructions and viral RNA was isolated from ATMUV infected CEF cells and control cellular RNA were prepared from uninfected CEF cells as previously described [33]. Approximately 2.0 × 10^6^ CEF cells per well in 6-well plates were transfected with 3 μg of VG-RNA, viral RNA and cellular RNA using Lipofectamine 2000 Transfection Reagent, respectively. The samples were examined by RT-PCR analysis 6 h after transfection.

### RT-PCR, quantitative real-time PCR

Total RNA was extracted from cells and spleens of chicks infected with ATMUV or SPF chick embryo allantoic fluid using Trizol reagent (TransGen Biotech, Beijing Co., Ltd) according to the manufacturer’s instructions. Equal amounts of RNA (4 μg) was used for reverse-transcription PCR to prepare cDNA using M-MLV Reverse Transcriptase (Promega, USA), followed by PCR using rTaq DNA polymerase and quantitative real-time PCR using TransStart Green qPCR SuperMix (TransGen). The primers for ATMUV and chicken IFN-β, IFN-λ gene were designed using the Primer 5 software; other sequences of the primers used in this study have been described previously [39–41]. All primers are shown in Table 1. The results were normalized using the housekeeping gene β-actin or GAPDH and analyzed as fold change relative to RNA samples from mock-infected samples.

**Table 1:** **Primers used in this study for RT-PCR and real time qRT-PCR**

Primer name	Sequence (5′-3′)		References
	Forward primer	Reverse primer	
ATMUV E	CCTACTGACACTGGGCATGG	AGGCAACCATCCTTTGTGCT	
Chicken β-actin	GCCAACAGAGAGAAGATGACAC	GTAACACCATCACCAGAGTCCA	[41]
Chicken IFN-α	ATGCCACCTTCTCTCACGAC	AGGCGCTGTAATCGTTGTCT	[41]
Chicken IFN-β	ACCAGGATGCCAACTTCT	TCACTGGGTGTTGAGACG	
Chicken IFN-λ	AGGATGAAGGAGCAGTTTGA	CCAGAGGGCTGATGTGAA	
Chicken TLR1	GGCAGTGGACGCAGACAAA	GTAGGAAATGAAGGCGTGGAA	[41]
Chicken TLR2	CTGAAGCCACAGACATTCCTAAC	CTTGTACCCAACGACCACCA	[41]
Chicken TLR3	GCAACACTTCATTGAATAGCCTTGAT	TTCAGTATAAGGCCAAACAGATTTCC	[41]
Chicken TLR4	GGCAAAAAATGGAATCACGA	CTGGAGGAAGGCAATCATCA	[41]
Chicken TLR5	TCAAAGATGGGTGGTGTGTAGAA	ACTGACGTTCCTTTGCACTTTTT	[41]
Chicken TLR7	ATGCTGTTATCAGGACGTTGGTT	CCTTGAGGCGACGGTCACT	[41]
Chicken TLR15	AACATCTACATCCGTAACCCG	TTAGCACCAGAACGACAAGG	[41]
Chicken TLR21	CAAGAAGCAGCGGGAGAAG	TCAGGATGCGGTTAAAGCG	[41]
Chicken MAD5	TGAAGGCAAAGAGAGATCAGCGTAAGA	CATATCAATTGTGGCAATTCTTGCACAGGA	[40]
Chicken IPS-1	GCAGTTTGATGCAGAGCAGAAGCA	AGGCTTCAAGGAGGTGTCACAGAA	[40]
Chicken Mx1	TTCACGTCAATGTCCCAGCTTTGC	ATTGCTCAGGCGTTTACTTGCTCC	[40]
Chicken OASL	GCAGAAGAACTTTGTGAAGTGGCG	TCGGCTTCAACATCTCCTTGTACC	[40]
Human GAPDH	AGAAGGCTGGGGCTCATTTG	AGGGGCCATCCACAGTCTTC	[39]
Human IFN-β	GCTCTCCTGTTGTGCTTCTCCAC	CAATAGTCTCATTCCAGCCAGTGC	
Human IL-28A/B	AGCTGCAGGCCTTTAAGAGG	TCCAGAACCTTCAGCGTCAG	[39]
Human IL-29	CCAAGCCCACCACAACTGGG	TCTGGTGCAGGGTGTGAAGG	
Human TLR3	TCACTTGCTCATTCTCCCTT	GACTCTCCATTCCTGGC	
Human MAD5	CTGCTGCAGAAAACAATGGA	TGGCTGAACTGTGGTTGAAA	
Human IRF3	CTGGGGCCCTTCATTGTAGA	TAGGCCTTGTACTGGTCGGA	
Human IRF7	CGAGACGAAACTTCCCGTCC	GCTGATCTCTCCAAGGAGCC	
Human IPS-1	TTCTCCTCCTCATCCCCTGG	GGATGGTGCTGGATTGGTGA	
Human Mx1	GACATTCGGCTGTTTACC	GCGGTTCTGTGGAGGTTA	
Human OASL	CCCTGAGGTCTATGTGAGC	GTGAAGCCTTCGTCCAAC	
Human OAS1	AGAGACTTCCTGAAGCAGCG	GAGCTCCAGGGCATACTGAG	
Human IFITM3	TGGCCAGCCCCCCAACTAT	CATAGGCCTGGAAGATCAG	

### TLR3-siRNA and generation of shRNA-based knockdown cell lines

The siRNA specifically targeting human TLR3 (TLR3-siRNA) and negative control siRNA (NC-siRNA) were purchased from Sangon Biotech Co., Ltd (Shanghai, China). The TLR3-siRNA sense sequence was: 5′-CCAACUCCUUUACAAGUUUTT-3′; Antisense: 5′-AAACUUGUAAAGGAGUUGGTT-3′. NC-siRNA sense sequence: 5′-UUCUCCGAACGUGUCACGUTT-3′; NC-siRNA antisense sequence: 5′-ACGUGACACGUUCGGAGAATT-3′. Cell lines stably expressing short hairpin RNA (shRNA) specifically targeting either MDA5, TLR3, IPS-1, IRF3, IRF7, or luciferase control were generated by infection of 293T cells with lentiviruses encoding these shRNA in pSIH-H1-GFP vector as previously described [33, 39, 42].

### Western blotting

Cell lysates were prepared, and Western blotting was performed as previously described [42]. Briefly, protein samples were fractionated by electrophoresis on 12% SDS polyacrylamide gels, transferred to nitrocellulose membranes, and then probed with appropriate dilutions of the indicated antibodies.

### Statistical analysis

The results are shown as mean values ± standard error (mean ± SE). Statistical significance was determined by the Student’s *t* test analysis. A level of *P* < 0.05 was considered to be significant.

## Results

### ATMUV infection induces robust expression of particular type I and type III IFN and some critical ISG in chicken embryo fibroblasts

**Figure 1:**
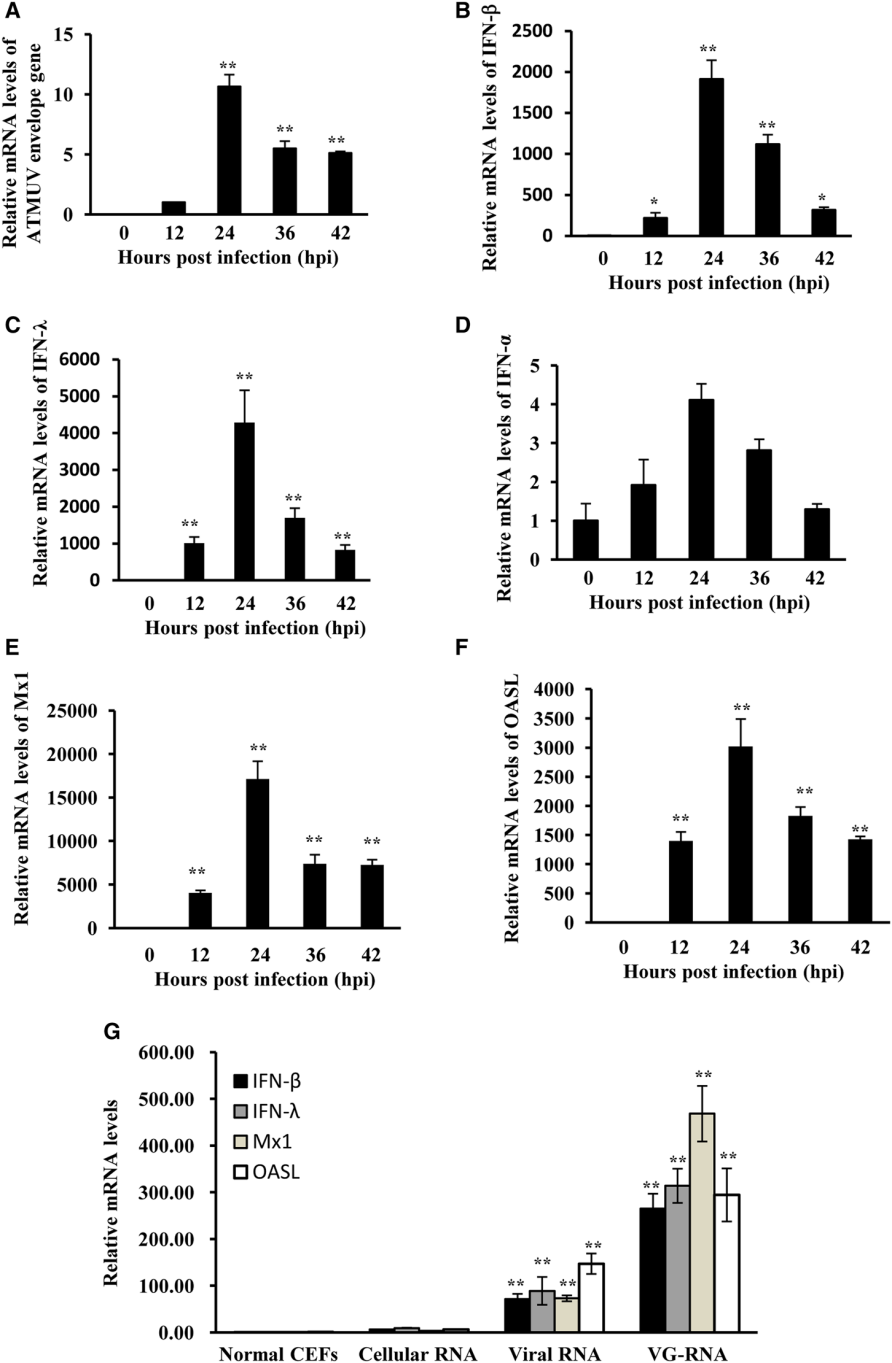
**ATMUV infection significantly up-regulated the expression of IFN and ISG in CEF cells.** Chicken embryo fibroblasts (CEF) were infected with or without ATMUV at a MOI of 1.0 and harvested at 0, 12, 24, 36 and 42 h post-infection (hpi). **A**–**F** Quantitative real-time PCR analysis was performed to examine the mRNA expression of ATMUV envelop gene (**A**), chicken type I  (**B**, **D**) and type III IFN (**C**), and key ISG Mx1 (**E**) and OASL (**F**). **G** ATMUV genomic RNA (VG-RNA), viral RNA or control cellular RNA were transfected into CEF cells for 6 h. The mRNA levels of IFN-β, IFN-λ, Mx1 and OASL were analyzed by quantitative real-time PCR. Expression of ATMUV envelop gene at 24–42 hpi was compared to its expression at 12 hpi. The mRNA expression of ATMUV envelop gene at 12 hpi was set to 1.0. The representatives of three independent experiments with similar results are shown. The average levels from three independent experiments are plotted. The error bars represent the S.E. Statistical significance of change was determined by the Student’s *t*-test (*P < 0.05, **P < 0.01).

To determine whether ATMUV infection could trigger host innate immune response, chicken embryo fibroblasts (CEF) were infected with ATMUV CJD05 strain at MOI of 1.0, harvested at different time points post infection, and examined for expression of ATMUV, IFN and ISG. Quantitative real-time PCR analysis shows that CEF cells could be easily infected, as evidenced by robust mRNA expression of ATMUV envelop protein gene (ATMUV E) (Figure 1A). Remarkably, we observed that the ATMUV infection greatly induced the expression of IFN-β and IFN-λ (Figures 1B and C). However, ATMUV infection had no significant effect on expression of IFN-α (Figure 1D). In addition, expression of two key ISG, Mx1 and OASL, was also examined by quantitative real time PCR during the ATMUV infection. Similarly, infection with ATMUV resulted in robust expression of Mx1 and OASL (Figures 1E and F). These observations were further confirmed by RT-PCR analysis (Additional file 1A). Taken together, these data suggest that ATMUV infection can trigger innate immune response in CEF.

To define the molecular basis of how ATMUV triggers host innate immune response, we investigated the PAMP of ATMUV that induced IFN expression. For this, ATMUV VG-RNA (RNA from the purified virus particles), viral RNA (RNA from ATMUV infected CEF cells) or control cellular RNA (RNA from uninfected CEF cells) were prepared and transfected into CEF cells using Lipofectamine 2000. Indeed, expression of IFN-β, IFN-λ, Mx1 and OASL was greatly up-regulated by transfection of CEF cells with either VG-RNA or viral RNA, whereas total RNA derived from normal control cells failed to stimulate IFN and ISG expression (Figure 1G; Additional file 1B). These results indicate that ATMUV genomic RNA serves as PAMP that is sufficient to induce host innate immune response.

### ATMUV infection triggers effectively innate immune response in chickens

**Figure 2:**
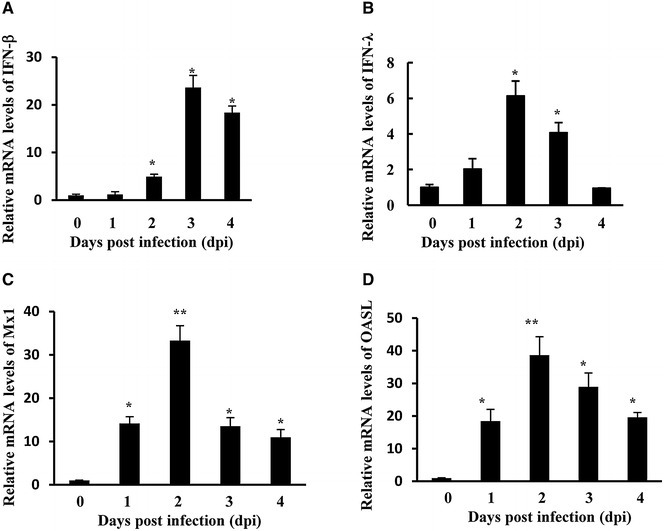
**The chicken innate immune response was induced by ATMUV infection.** Each young SPF chick was challenged by intramuscular inoculation with 4.0 × 10^5^ EID_50_ of ATMUV in a volume of 0.4 mL. **A**–**D** The spleen tissues of mock and ATMUV infected chicks were collected at the time indicated for examination of IFN-β (**A**), IFN-λ (**B**) and ISG (**C**, **D**) mRNA expressions using quantitative real-time PCR. The average levels from three independent experiments are plotted. The error bars represent the S.E. *P < 0.05, **P < 0.01.

Next, we asked whether ATMUV infection could induce host innate immune response in vivo. To this end, each 5-day-old SPF chick was inoculated intramuscularly with 4.0 × 10^5^ EID_50_ of CJD05 in a volume of 0.4 mL. Spleens of mock or ATMUV infected chicks were collected at the indicated time to examine the expression of IFN and ISG (Figures 2A–D), since previous studies have shown that the highest ATMUV titer was detected in the spleen of infected animals as compared to other parenchymatous organs [43]. Interestingly, analysis of quantitative real-time PCR showed that mRNA levels of IFN-β was gradually elevated from day 1 to day 3 post infection and then declined (Figure 2A). Expression of IFN-λ and some key ISG (OASL and Mx1) was induced in the early time and reached at their maximum value on day 2 post infection, and then started to decline gradually from day 3 post infection (Figures 2B–D). The mRNA expression of IFN-β, IFN-λ, OASL and Mx1 was further confirmed by RT-PCR (Additional file 2). These results provide strong evidence that host innate immune response can be triggered by ATMUV infection in vivo.

### ATMUV infection causes robust expression of type I and type III IFN and ISG in human 293T cells

**Figure 3:**
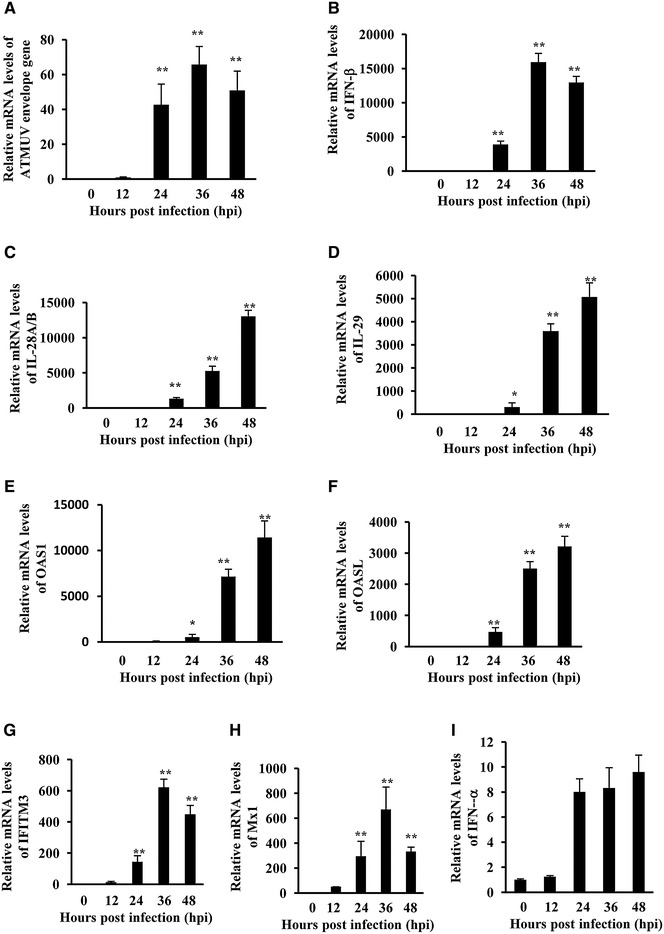
**Robust expression of particular IFN and ISG was also induced in human 293T cells during the ATMUV infection.** 293T cells were infected with or without ATMUV at an MOI of 1.0 and harvested at 0, 12, 24, 36 and 48 hpi, respectively. Quantitative real-time PCR analysis was performed to examine the mRNA expression of ATMUV envelop gene (**A**), human type I and type III IFN (**B**–**D**, **I**) and indicated key ISG (**E**–**H**). ATMUV envelop gene’s mRNA expression at 12 hpi was set to 1.0. ATMUV envelop gene expression at 24–42 hpi was compared to the expression at 12 hpi. The average levels from three independent experiments are plotted. The error bars represent the S.E. *P < 0.05, **P < 0.01.

To further understand the molecular basis about regulation of IFN response, we wished to establish a model cell system for the analysis of functional involvement of signaling pathways in innate immunity during ATMUV infection, because CEF have difficult survival after several passages. Thus, the human 293T cell line was employed. As expected, the 293T cells were easily infected with ATMUV after several passage subcultivation (Figure 3A; Additional file 3), consistent with previous reports indicating that humans could be infected by ATMUV [6, 7]. Importantly, expression of IFN-β, IL-28A/B, IL-29 was greatly induced by ATMUV infection as compared to mock treatment (Figures 3B–D; Additional file 3). Similarly, expression levels of some key ISG, including OAS1, OASL, IFITM3 and Mx1 were also significantly elevated (Figures 3E–H; Additional file 3). However, IFN-α expression was only slightly increased (Figure 3I; Additional file 3). These data are consistent with the observations in CEF cells. Together, these experiments suggest that the 293T cell line exhibited similar innate immune response to ATMUV infection as CEF did, and thus was selected as a model cell in this study.

### ATMUV infection triggers innate immune response via MDA5 and TLR3-dependent signaling pathways

**Figure 4:**
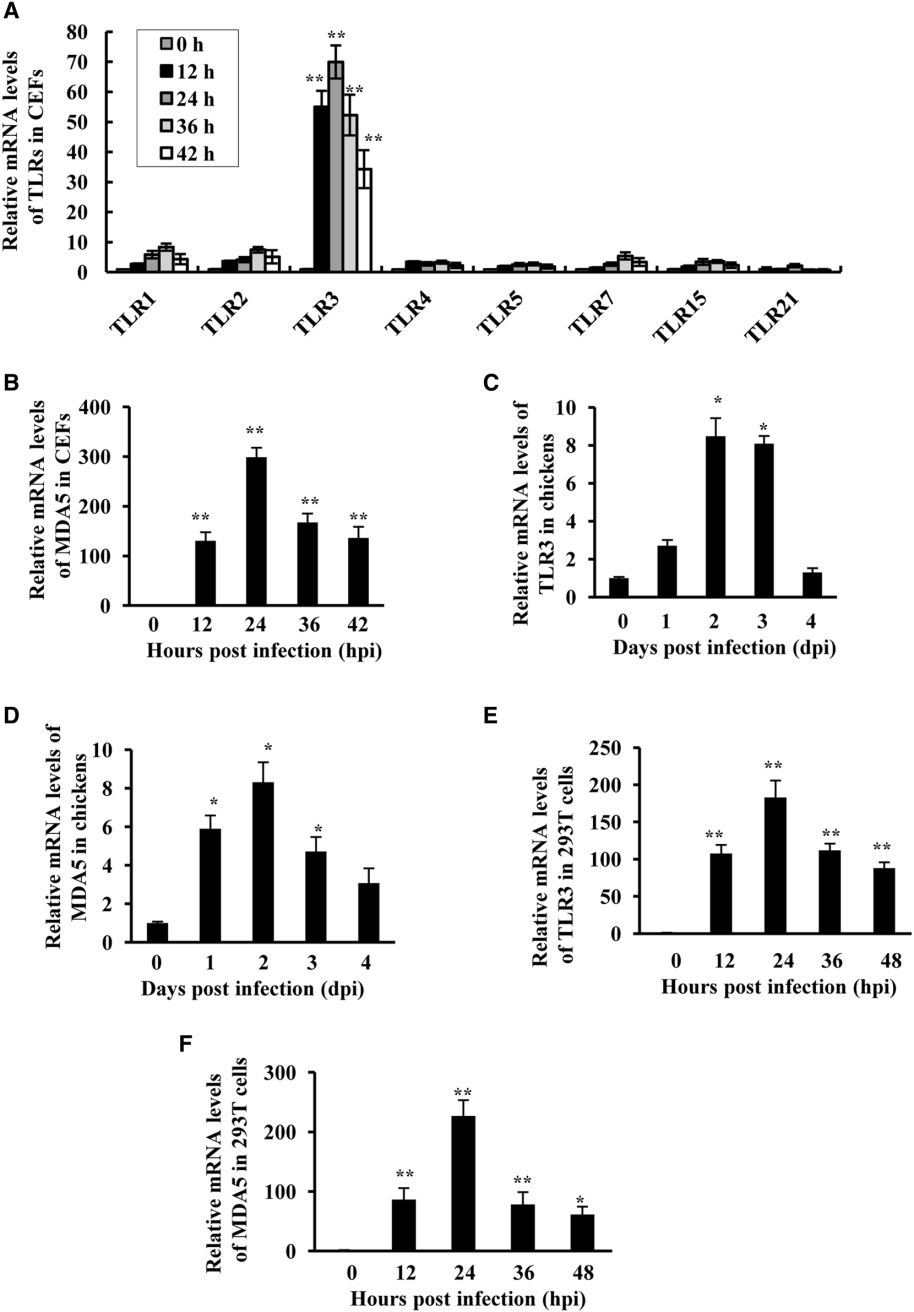
**ATMUV infection causes significant up-regulation of TLR3 and MDA5. A** Quantitative real-time PCR was performed to examine the mRNA expression of TLR1, TLR2, TLR3, TLR5, TLR7, TLR15 and TLR21 in CEF cells after ATMUV infection. **B** mRNA levels of MDA5 in ATMUV infected CEF cells were analysed by quantitative real-time PCR. **C**, **D** The spleen tissues derived from ATMUV infected chicks were analysed for mRNA expression of MDA5 and TLR3 by quantitative real-time PCR. **E**, **F** mRNA expression of MDA5 and TLR3 in ATMUV infected 293T cells was analysed by quantitative real-time PCR. The average levels from three independent experiments are plotted. The error bars represent the S.E. *P < 0.05, **P < 0.01.

Intracellular detection of viral infections by PRR activates the innate immune signaling. Therefore, we determined which PRR are involved in sensing ATMUV infection. To this end, CEF, chicks and 293T cell lines were infected with or without ATMUV and harvested at different time points, followed by quantitative real-time PCR analysis of PRR, except RIG-I which in previous investigations was identified as being absent in chickens [44]. As shown in Figures  4A and B, ATMUV infection resulted in significantly increased expression of TLR3 and MDA5, but had little effect on the expression of TLR1, TLR2, TLR5, TLR7, TLR15 and TLR21 in CEF cells. Similarly, the mRNA levels of TRL3 and MDA5 were also elevated in ATMUV infected chickens (Figures 4C and D) and 293T cells (Figures 4E and F). Consistent with these observations, RT-PCR analysis also exhibited the increase in expression of TLR3 and MDA5 after ATMUV infection (Additional files 4A–C).

**Figure 5:**
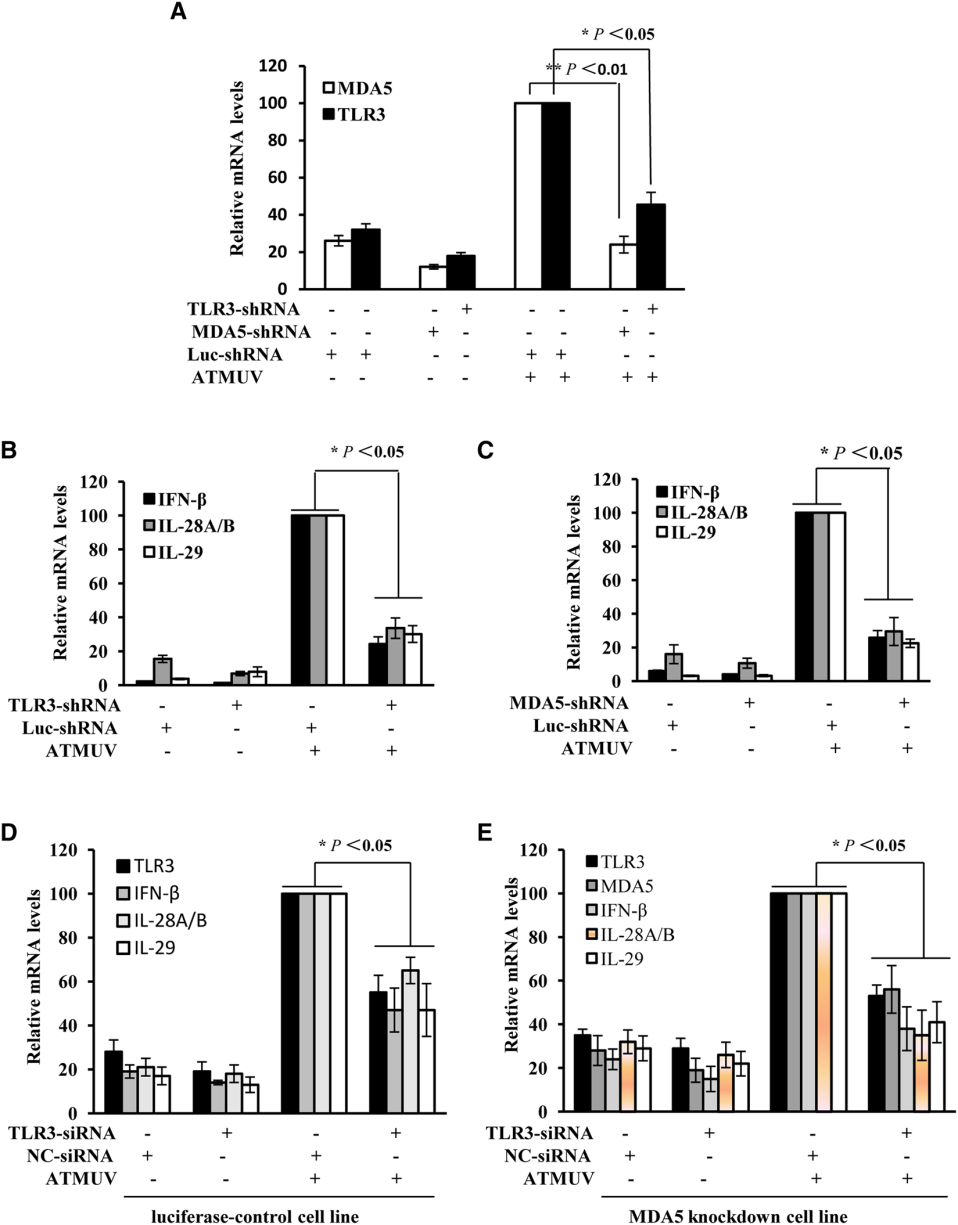
**ATMUV infection triggers innate immune response via MDA5 and TLR3-dependent signaling pathways.** 293T cells stably expressing shRNA specifically targeting either TLR3, MDA5 or luciferase control were infected with or without ATMUV for 36 h. Quantitative real-time PCR were then performed to determine the interference efficiency of TLR3, MDA5 (**A**) and examine the IFN expression in 293T cells stably expressing shRNA targeting TLR3 or MDA5 (**B**, **C**). **D** and **E** 100 nM TLR3-siRNA or NC-siRNA was transfected into MDA5-knockdown or luciferase-knockdown 293T cell lines, interference efficiency of TLR3, MDA5 and IFN expression were analysed by quantitative real-time PCR. The mRNA level in ATMUV infected 293T cells expressing luc-shRNA control was set to 100. The average levels from three independent experiments are plotted. The error bars represent the S.E. *P < 0.05, **P < 0.01.

To investigate the functional involvement of TRL3 and MDA5 in innate immunity during ATMUV infection, we generated 293T cell lines stably expressing specific shRNA targeting either TLR3, MDA5 or luciferase control. These cells were then infected with or without ATMUV for 36 h, and interference efficiency of the shRNA and their effects on IFN expression were examined by quantitative real-time PCR. As shown in Figure 5A, expression of TLR3 and MDA5 were significantly knocked down by the specific shRNA. Importantly, we observed that disruption of TLR3 or MDA5 expression resulted in a significant decrease in mRNA levels of IFN-β, IL-28A/B and IL-29 induced by ATMUV infection (Figures 5B and C and Additional files 5A, B).

Furthermore, we examined an effect of silencing simultaneously both MDA5 and TLR3 on the expression of IFN in 293T cells. For this, 100 nM TLR3-siRNA or NC-siRNA was transfected into MDA5-knockdown or luciferase-knockdown 293T cell lines (approximately 1.0 × 10^6^ cells/well). Twenty-four hours post transfection, the cells were infected with ATMUV at the MOI of 1.0 and harvested at 36 h post infection. Strikingly, transfection of TLR3-siRNA and MDA5 shRNA significantly disrupted the expression of both TLR3 and MDA5, associated with lower mRNA levels of IFN-β, IL-28A/B, IL-29 after ATMUV infection than those observed in cells silencing MDA5 only (Figures 5D and E). These data indicate that both TLR3 and MDA5 are implicated in host IFN response to ATMUV infection.

### IPS-1 plays an essential role in ATMUV-induced up-regulation of IFN

**Figure 6:**
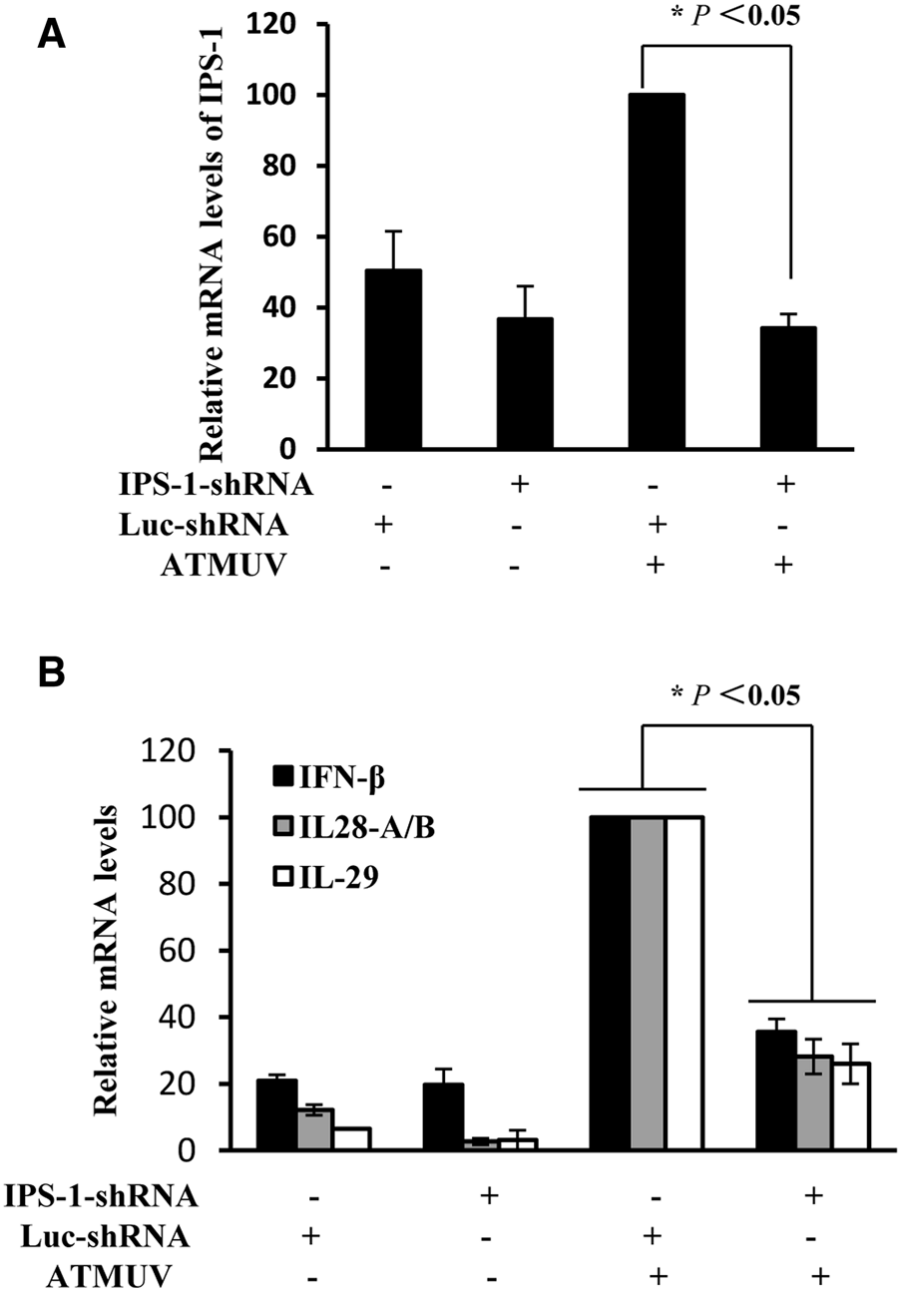
**IPS-1 plays an essential role in ATMUV-induced up-regulation of IFN.** 293T cells stably expressing specific shRNA targeting IPS-1 or luciferase control were infected with or without ATMUV for 36 h. Quantitative real-time PCR were performed to measure the interference efficiency of IPS-1 (**A**) and the production of IFN-β, IL-28A/B and IL-29 (**B**). The mRNA level in the luc-shRNA control cells infected with ATMUV was set to 100. The average results from three independent experiments are plotted. The error bars represent the S.E. *P < 0.05.

IPS-1, a key cellular adaptor protein, is required for MDA5-dependent innate immune signaling [45, 46]. Next, we investigated whether the IPS-1-dependent pathway is essential for the innate immune response during ATMUV infection. To address this issue, 293T cell line stably expressing shRNA specifically targeting IPS-1 was generated and subsequently, interference efficiency of IPS-1 was determined upon ATMUV infection. Quantitative real-time PCR experiments show that the level of IPS-1 was significantly reduced following expression of the shRNA (Figure 6A). Indeed, expression of IFN-β, IL-28A/B and IL-29 was significantly suppressed in IPS-1 knockdown cells after infection with ATMUV as compared to the luciferase control (Figure 6B and Additional file 6). These observations suggest that IPS-1 is involved in regulation of ATMUV-induced expression of IFN.

### IRF3, IRF7 and NF-κB are required for efficient expression of IFN induced by ATMUV

**Figure 7:**
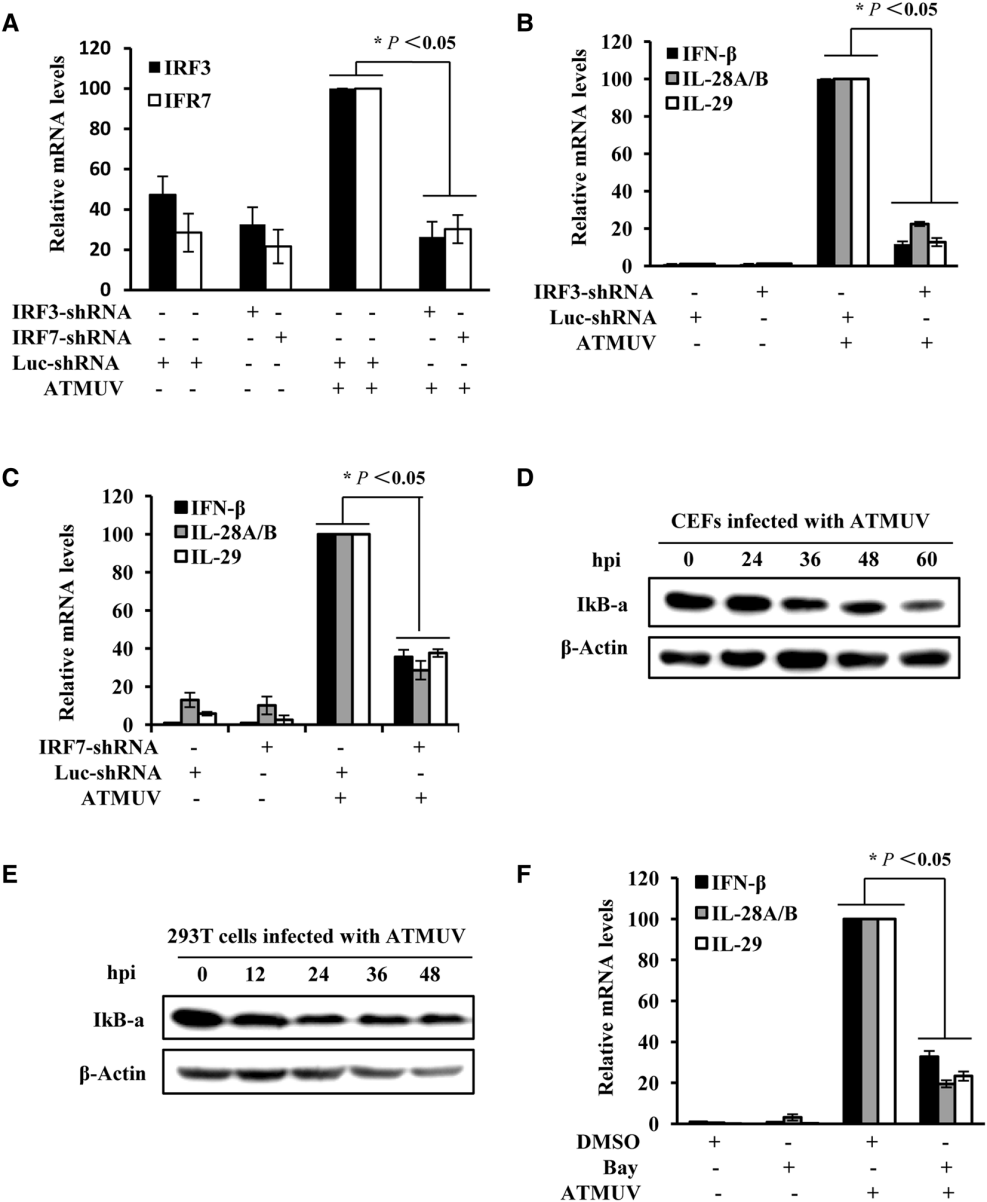
**IRF3, IRF7 and NF-κB activation are required for efficient expression of IFN induced by ATMUV.** 293T cells stably expressing specific shRNA targeting IRF3, IRF7 or luciferase control were infected with or without ATMUV for 36 h. Quantitative real-time PCR were performed to measure the interference efficiency of IRF3, IRF7 (**A**) and the production of IFN-β, IL-28A/B and IL-29 (**B**, **C**). CEF (**D**) and 293T (**E**) cells infected with ATMUV for the indicated times were harvested and protein expression of IκB-α was examined by Western blotting. **F** 293T cells were treated with BAY11-7082 (5 µM) or DMSO for 3 h, followed by ATMUV infection for 36 h infection. The cells were harvested and the mRNA levels of IFN-β, IL-28A/B and IL-29 were analyzed by quantitative real-time PCR. Shown are representatives of three independent experiments with similar results. The mRNA level of positive control cells was set to 100. The average results from three independent experiments are plotted. The error bars represent the S.E. *P < 0.05, **P < 0.01.

It is well known that transcription of innate immune molecules is dependent on the activation of several transcriptional factors such as the nuclear factor κB (NF-κB) and IFN regulatory factors (IRF) [18, 32, 47]. In addition, it is thought that IRF3 and IRF7 can be activated by TLR-dependent signaling pathway and regulate type I IFN response [38]. Thus, we asked whether these transcription factors govern the IFN production during the ATMUV infection. To test this possibility, shRNA-based knockdown of IRF3 and IRF7 was performed in 293T cells (Figure 7A). We found that expression of type I and III IFN (IFN-β, IL-28A/B and IL-29) was significantly inhibited by silencing IRF3 and IRF7 in ATMUV-infected cells (Figures 7B and C; Additional files 7A and B).

NF-κB is a major transcription factor that regulates genes responsible for a variety of immune responses [48]. In non-stimulated cells, the NF-κB dimmers are sequestered in the cytoplasm by IκB. Degradation of IκB causes activation of NF-κB that enters the nucleus where it turns on the transcription of targeting genes. To evaluate the role of NF-κB in response to ATMUV infection, the protein expression of IκB-α was examined by Western blotting. We observed that IκB-α protein levels were consistently reduced in CEF and 293T cells infected with ATMUV as compared to mock control, suggesting that NF-κB is activated during the ATMUV infection (Figures 7D and E). To further determine the functional relevance of NF-κB, 293T cells were treated with either BAY11-7082, an inhibitor of NF-κB, or DMSO for 3 h, followed by ATMUV infection. As expected, expression of IFN-β, IL-28A/B and IL-29 was clearly inhibited by inactivation of NF-κB in cells infected with ATMUV (Figure 6F and Additional file 7C). Together, these data reveal that transcription factors IRF3, IRF7 and NF-κB play important roles in regulating the type I and type III IFN production in response to ATMUV infection.

### Pretreatment of host cells with IFN significantly impairs replication of ATMUV

**Figure 8:**
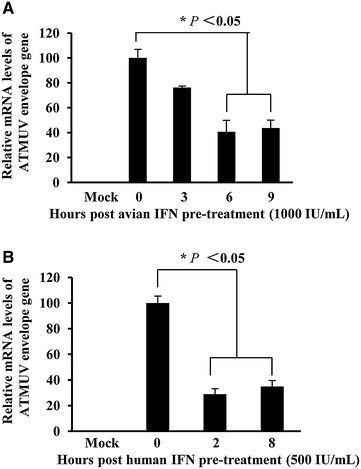
**Pretreatment of host cells with IFN significantly impairs replication of ATMUV. A** CEF cells were incubated with avian IFN (1000 IU/mL) for the indicated time before ATMUV infection at a MOI of 0.1. Quantitative real-time PCR were performed to examine expression of ATMUV envelop gene. **B** 293T cells were pretreated with human IFN-β (500 IU/mL) for the indicated time before infection with ATMUV at a MOI of 0.1. Expression of ATMUV envelop gene was examined by quantitative real-time PCR. The viral mRNA level in control cells (no IFN pretreatment cells) after ATMUV infection was set to 100. The virus suppression efficiency in IFN pretreatment cells was compared to the control. The average results from three independent experiments are plotted. The error bars represent the S.E. *P < 0.05.

Previous studies have demonstrated that type I IFN had an inhibitory effect on infection of CCHFV and dengue virus [49, 50]. Because our data revealed that ATMUV infection caused great up-regulation of type I and type III IFN, we tested whether these IFN had antiviral activity in response to ATMUV infection. For this, CEF cells were incubated with avian IFN at the concentration of 1000 IU/mL for 3, 6 and 9 h respectively, and then infected with ATMUV at a MOI of 0.1 for 24 h. We found that replication of ATMUV is significantly impaired by treatment with avian IFN (Figure 8A; Additional file 8A). To confirm this finding, 293T cells were incubated with human IFN-β (500 IU/mL) for 2 and 8 h, and then infected with ATMUV at a MOI of 0.1 for 24 h. Similarly, pretreatment of 293T cells with IFN-β significantly inhibited the replication of ATMUV (Figure 8B; Additional file 8B). Taken together, these experiments indicate that ATMUV infection activates host innate immune signaling and induces an effective antiviral immune response involving several critical IFN.

## Discussion

Although ATMUV was identified a long time ago, its pathogenesis is poorly understood. Sitiawan virus, a broiler-origin ATMUV, was the first strain of ATMUV that was shown to cause encephalitis and retard growth of chicks in 2000 [51]. Since 2009, the Chinese ATMUV strain has become highly pathogenic to domestic chickens, ducks and geese with symptoms characterized by a severe egg drop and neurological syndrome [1, 2, 4]. Over the past several years, studies have been focused on ATMUV isolation, identification, genomic sequencing, diagnosis and clinical investigations [52–55]. However, the molecular mechanism underlying interaction between ATMUV and its host remains to be determined. Host innate immunity is the first line of defense against pathogen infection. This includes production of various IFN and hundreds of ISG. In a previous study, ATMUV strain JD05 was characterized as a highly pathogenic virus to chickens and ducklings, but its pathogenesis is still not clear [1]. In this study, we explored host innate immune response following ATMUV infection. Our data establish that ATMUV infection can effectively activate host innate immune signaling and cause robust expression of several critical IFN and ISG.

Chinese ATMUV was originally isolated from sick chickens or ducks, but viral RNA and antibodies were also detected in poultry workers [7]. ATMUV has already evolved to cross the species barrier and shows a potential threat to humans. Previous investigations have observed ducklings’ immune response to ATMUV infection [43, 56]. However, little is known about human and chicken innate immunity against ATMUV infection. In the present study, we found ATMUV infection can effectively activate both MDA5 and TLR3 mRNA up-regulation in CEF cells, 293T cells and chickens. Interestingly, human 293T cells exhibited similar innate immune response to ATMUV infection as CEF did (such as PRR and interferon up-expression). Because we have previously generated 293T cell lines stably expressing specific shRNA targeting either MDA5, RIG-I, TLR3, IPS-1, IRF3, IRF7, or luciferase control [33, 39, 42], 293T cell was selected as a model cell system to perform experimentation in this study. Our results reveal that both TLR3 and MDA5 are involved in host innate immune response to ATMUV infection. Although RIG-I is absent in chickens, chicken MDA5 might compensate for RIG-I’s function.

Different PRR recognize different microbial components and play differential roles in host antiviral defense [57]. It has been shown that viral double-stranded RNA (dsRNA) is a ligand for TLR3 and viral single-stranded RNA (ssRNA) is a ligand for TLR7/8 [13]. Indeed, in this study, we observed that the mRNA level of TLR3 is greatly up-regulated in the ATMUV-infected host, whereas no significant effect of ATMUV was seen on expression of other TLR including TLR1, TLR2, TLR4, TLR5, TLR7, TLR15 and TLR21. Similarly, ATMUV infection also elevated the expression of MDA5 in the host. Furthermore, silencing MDA5 and TLR3 significantly reduced the production of IFN. These results provide strong evidence that sensing ATMUV infection by MDA5 and TLR3 is critical for innate immune response during this virus infection. Previous reports showed that TLR3, MDA5 and RIG-I are involved in intracellular detection of dengue virus infection [58]. Because chicken lacks the RIG-I [44], it is still unclear whether RIG-I plays a role in sensing ATMUV infection in other hosts. This remains to be further defined.

IRF3, IRF7 and NF-κB are key transcription factors that regulate expression of type I IFN and downstream effectors of ISG. In this study, our experiments demonstrate for the first time that these transcription factors are also important in regulating the expression of type III IFN during the ATMUV infection. The non-redundant roles of IRF3 and IRF7 have been documented in pathogenesis of other viruses [32, 46]. For example, the roles of IRF3 and IRF7 in innate antiviral immunity against infection of West Nile Virus and dengue virus have been verified in previous studies [7, 59, 60]. Consistent with these observations, we found that disruption of IRF3 and IRF7 or inactivation of NF-κB significantly reduces type I and type III IFN production induced by ATMUV. These data suggest that ATMUV may trigger the same innate immune signaling pathways as other flavivirus do. However, further studies are needed to address whether other transcription factors (such as IRF1 and IRF5) play roles in innate immunity against ATMUV infection.

Interferons play a vital role in the early antiviral response [23]. It has been shown that IFN pretreatment of the host could inhibit dengue viral replication [50]. However, the effectiveness of IFN in countering ATMUV infection has not been determined. Here, our data suggest that both avian and human cells pretreated with specific IFN can successfully suppress the replication of ATMUV. These experiments suggest that ATMUV infection activates host innate immune signaling and induces an effective antiviral immune response through production of IFN, and provide a useful line of evidence for preventing and controlling ATMUV infection using IFN in the future.

In summary, our results establish for the first time that ATMUV infection triggers effectively IFN response through MDA5 and TLR3-dependent signaling pathways involving IPS-1, IRF3, IRF7 and NF-κB. In addition, our experiments provide evidence that IFN response functions effectively suppress the replication of ATMUV. However, further investigations are still required to address the molecular mechanisms underlying complex interaction between ATMUV and the host, including how the viral non structural protein(s) antagonizes IFN response and overcomes the host innate immunity.

## Additional files

10.1186/s13567-016-0358-5 **The expression of IFN and ISG were significantly up-regulated after ATMUV infected CEF cells.** A. CEF were infected with ATMUV and harvested at 0, 12, 24, 36 and 42 hpi. RT-PCR analysis was performed to examine the mRNA expression of ATMUV envelop gene, chicken type I and type III IFN and key ISG Mx1 and OASL. B. ATMUV genomic RNA (VG-RNA), viral RNA or control cellular RNA were transfected into CEF cells for 6 h. The mRNA levels of IFN-β, IFN-λ, Mx1 and OASL were analyzed by RT-PCR.
10.1186/s13567-016-0358-5 **Chicken innate immune response was induced by ATMUV infection.** Each young SPF chick was challenged by intramuscular inoculation with 4.0 × 10^5^ EID_50_ of ATMUV in a volume of 0.4 mL. The spleen tissues were collected at the indicated time for examination of the mRNA expression of IFN-β, IFN-λ and ISG using RT-PCR.
10.1186/s13567-016-0358-5 **Robust expression of particular IFN and ISG was also induced in human 293T cells during the ATMUV infection.** 293T cells were infected with or without ATMUV at a MOI of 1.0 and harvested at 0, 12, 24, 36 and 48 hpi, respectively. RT-PCR analysis was performed to examine the mRNA expression of the ATMUV envelop gene, human type I and type III IFN and indicated key ISG.
10.1186/s13567-016-0358-5 **ATMUV infection causes significant up-regulation of TLR3 and MDA5.** RT-PCR was performed to examine the mRNA expression of TLR3 and MDA5 in CEF (A), chickens (B) and 293T cells (C) at the indicated time after ATMUV infection, respectively.
10.1186/s13567-016-0358-5 **ATMUV infection triggers innate immune response via MDA5 and TLR3-dependent signaling pathways.** RT-PCR were performed to examine the IFN expression and the interference efficiency of TLR3 or MDA5 in TLR3 (A) or MDA5 (B) knockdown 293T cell lines after ATMUV infection for 36 h.
10.1186/s13567-016-0358-5 **IPS-1 plays an essential role in ATMUV-induced up-regulation of IFN.** IPS-1 knockdown 293T cell line or luciferase control were infected with or without ATMUV for 36 h. RT-PCR were performed to measure the interference efficiency of IPS-1 and the production of IFN-β, IL-28A/B and IL-29.
10.1186/s13567-016-0358-5 **IRF3, IRF7 and NF-κB activation are required for efficient expression of IFN induced by ATMUV.** A and B. IRF3, IRF7 knockdown 293T cell lines or luciferase control were infected with or without ATMUV for 36 h. RT-PCR were performed to measure the interference efficiency of IRF3, IRF7 and the production of IFN-β, IL-28A/B and IL-29. C. 293T cells were treated with BAY11-7082 (5 µM) or DMSO for 3 h, followed by ATMUV infection for 36 h. The cells were harvested and the mRNA levels of IFN-β, IL-28A/B and IL-29 were analyzed by RT-PCR.
10.1186/s13567-016-0358-5 **Pretreatment of host cells with IFN significantly impairs replication of ATMUV.** A. CEF cells were incubated with avian IFN (1000 IU/mL) for the indicated time before ATMUV infection. RT-PCR were performed to examine expression of ATMUV envelop gene. B. 293T cells were pretreated with human IFN-β (500 IU/mL) for the indicated time before infection with ATMUV. Expression of ATMUV envelop gene was examined by RT-PCR.
