# Supplementary figures and images for: Avian Tembusu virus infection effectively triggers host innate immune response through MDA5 and TLR3-dependent signaling pathways
Source: Vet Res. 2016 Jul 22;47:74. doi: 10.1186/s13567-016-0358-5 (PMC4957414; doi:10.1186/s13567-016-0358-5)

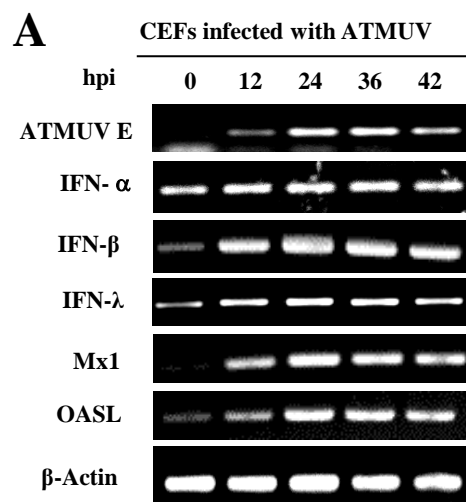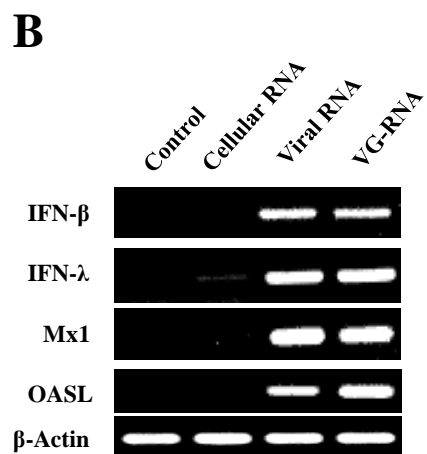

Supplement: Supplementary file 1 — 10.1186/s13567-016-0358-5 The expression of IFN and ISG were significantly up-regulated after ATMUV infected CEF cells. A. CEF were infected with ATMUV and harvested at 0, 12, 24, 36 and 42 hpi. RT-PCR analysis was performed to examine the mRNA expression of ATMUV envelop gene, chicken type I and type III IFN and key ISG Mx1 and OASL. B. ATMUV genomic RNA (VG-RNA), viral RNA or control cellular RNA were transfected into CEF cells for 6 h. The mRNA levels of IFN-β, IFN-λ, Mx1 and OASL were analyzed by RT-PCR. [file 13567_2016_358_MOESM1_ESM.pdf]

Spleen of chicken infected with ATMUV

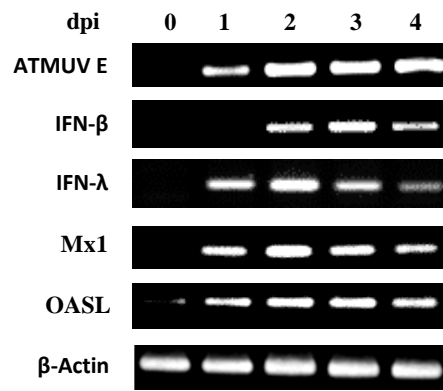

Supplement: Supplementary file 2 — 10.1186/s13567-016-0358-5 Chicken innate immune response was induced by ATMUV infection. Each young SPF chick was challenged by intramuscular inoculation with 4.0 × 10^5^ EID50 of ATMUV in a volume of 0.4 mL. The spleen tissues were collected at the indicated time for examination of the mRNA expression of IFN-β, IFN-λ and ISG using RT-PCR. [file 13567_2016_358_MOESM2_ESM.pdf]

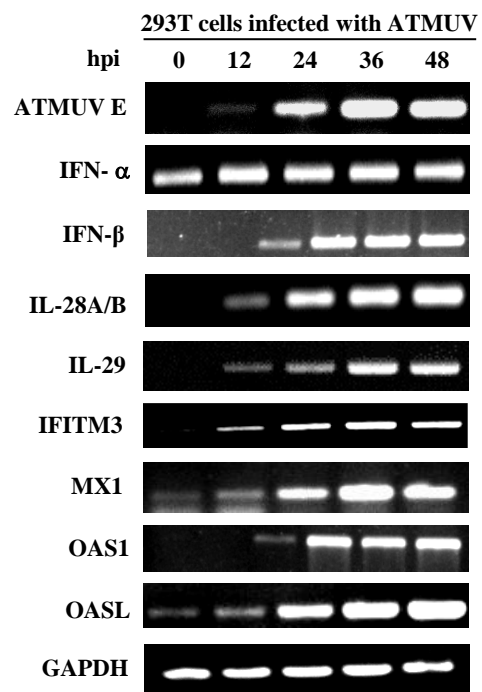

Supplement: Supplementary file 3 — 10.1186/s13567-016-0358-5 Robust expression of particular IFN and ISG was also induced in human 293T cells during the ATMUV infection. 293T cells were infected with or without ATMUV at a MOI of 1.0 and harvested at 0, 12, 24, 36 and 48 hpi, respectively. RT-PCR analysis was performed to examine the mRNA expression of the ATMUV envelop gene, human type I and type III IFN and indicated key ISG. [file 13567_2016_358_MOESM3_ESM.pdf]

**A**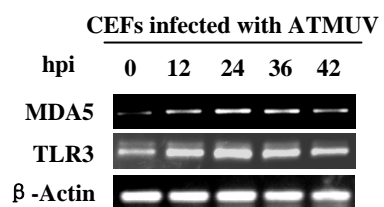**B**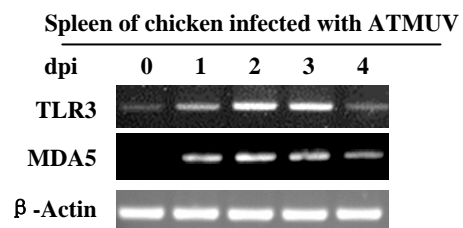**C**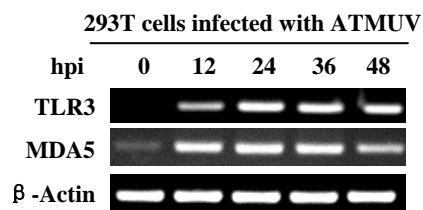

Supplement: Supplementary file 4 — 10.1186/s13567-016-0358-5 ATMUV infection causes significant up-regulation of TLR3 and MDA5. RT-PCR was performed to examine the mRNA expression of TLR3 and MDA5 in CEF (A), chickens (B) and 293T cells (C) at the indicated time after ATMUV infection, respectively. [file 13567_2016_358_MOESM4_ESM.pdf]

A

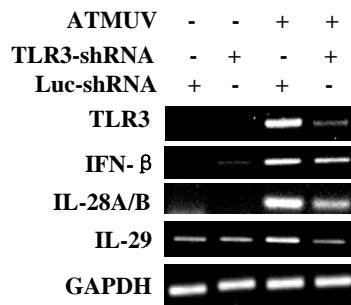

B

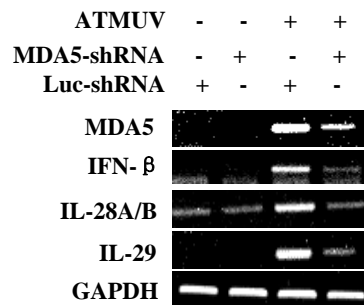

Supplement: Supplementary file 5 — 10.1186/s13567-016-0358-5 ATMUV infection triggers innate immune response via MDA5 and TLR3-dependent signaling pathways. RT-PCR were performed to examine the IFN expression and the interference efficiency of TLR3 or MDA5 in TLR3 (A) or MDA5 (B) knockdown 293T cell lines after ATMUV infection for 36 h. [file 13567_2016_358_MOESM5_ESM.pdf]

|             |   |   |   |   |
|-------------|---|---|---|---|
| ATMUV       | - | - | + | + |
| IPS-1-shRNA | - | + | - | + |
| Luc-shRNA   | + | - | + | - |

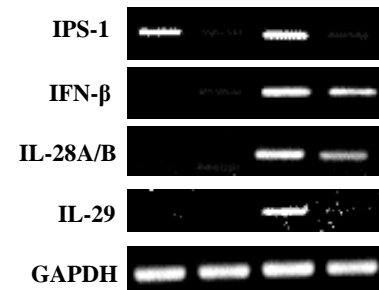

Supplement: Supplementary file 6 — 10.1186/s13567-016-0358-5 IPS-1 plays an essential role in ATMUV-induced up-regulation of IFN. IPS-1 knockdown 293T cell line or luciferase control were infected with or without ATMUV for 36 h. RT-PCR were performed to measure the interference efficiency of IPS-1 and the production of IFN-β, IL-28A/B and IL-29. [file 13567_2016_358_MOESM6_ESM.pdf]

**A**

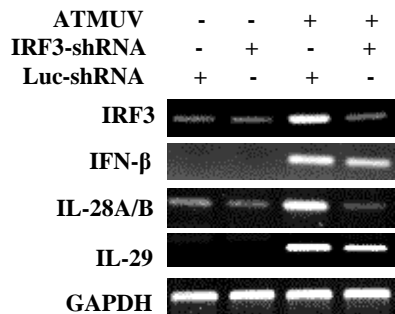

**B**

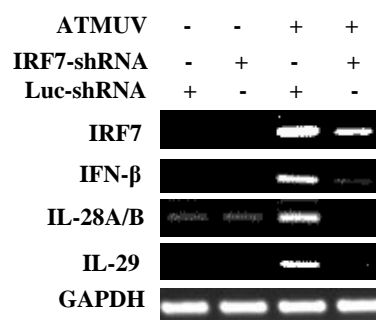

**C**

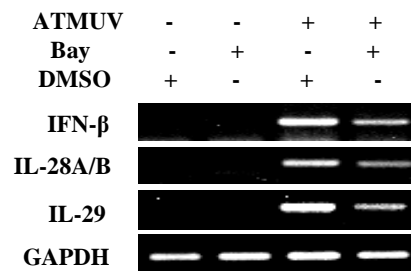

Supplement: Supplementary file 7 — 10.1186/s13567-016-0358-5 IRF3, IRF7 and NF-κB activation are required for efficient expression of IFN induced by ATMUV. A and B. IRF3, IRF7 knockdown 293T cell lines or luciferase control were infected with or without ATMUV for 36 h. RT-PCR were performed to measure the interference efficiency of IRF3, IRF7 and the production of IFN-β, IL-28A/B and IL-29. C. 293T cells were treated with BAY11-7082 (5 µM) or DMSO for 3 h, followed by ATMUV infection for 36 h. The cells were harvested and the mRNA levels of IFN-β, IL-28A/B and IL-29 were analyzed by RT-PCR. [file 13567_2016_358_MOESM7_ESM.pdf]

**A**

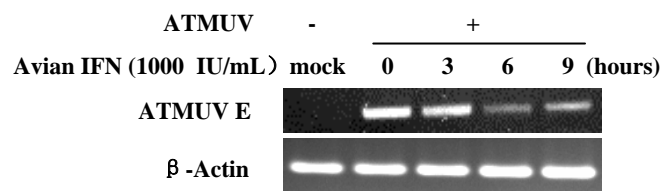

**B**

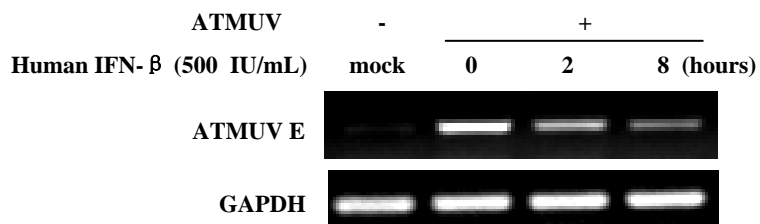

Supplement: Supplementary file 8 — 10.1186/s13567-016-0358-5 Pretreatment of host cells with IFN significantly impairs replication of ATMUV. A. CEF cells were incubated with avian IFN (1000 IU/mL) for the indicated time before ATMUV infection. RT-PCR were performed to examine expression of ATMUV envelop gene. B. 293T cells were pretreated with human IFN-β (500 IU/mL) for the indicated time before infection with ATMUV. Expression of ATMUV envelop gene was examined by RT-PCR. [file 13567_2016_358_MOESM8_ESM.pdf]
